# A Metabolism-Based Synergy for Total Coumarin Extract of Radix *Angelicae Dahuricae* and Ligustrazine on Migraine Treatment in Rats

**DOI:** 10.3390/molecules23051004

**Published:** 2018-04-25

**Authors:** Shan Feng, Xin He, Peiru Zhong, Jinyi Zhao, Cong Huang, Zhuohan Hu

**Affiliations:** 1 College of Pharmaceutical Sciences and Chinese Medicine, Southwest University, 2# Tiansheng Road, Beibei District, Chongqing 400715, China; fengshan@swu.edu.cn; 2 School of Chinese Materia Medica, Tianjin University of Traditional Chinese Medicine, 312# Anshanxi Road, Nankai District, Tianjin 300193, China; zhongpeiru@126.com (P.Z.); tingfengyusheng@163.com (J.Z.); huangcong236@sina.com (C.H.); 3 Tianjin State Key Laboratory of Modern Chinese Medicine, 88# Yuquan Road, Nankai District, Tianjin 300193, China; 4 Research Institute for Liver Diseases (Shanghai) Co. Ltd., 200# Niudun Road, Shanghai 201203, China; huzh@rild-biotech.com

**Keywords:** CYP 450s, ligustrazine, migraine, Radix *Angelicae dahuricae*, synergy

## Abstract

Radix *Angelicae dahuricae*, containing coumarins, which might affect cytochrome P450 enzyme (CYP450) activity, has been co-administered with ligustrazine, a substrate of CYP450s, for the clinical treatment of migraine. However, whether a pharmacokinetic-based synergy exists between Radix *Angelicae dahuricae* and ligustrazine is still unknown. In this study, the total coumarin extract (TCE) of Radix *Angelicae dahuricae* (50 mg/kg, orally) reinforced the anti-migraine activity of ligustrazine by declining head scratching, plasma calcitonin gene-related peptide, and serum nitric oxide, as well as increasing plasma endothelin levels in rats (*p* < 0.05). Moreover, the pharmacokinetic study reflected that TCE potentiated the area under the concentration–time curve of ligustrazine and prolonged its mean retention time in rats (*p* < 0.05). Besides, the IC_50_ for TCE, imperatorin and isoimperatorin inhibiting ligustrazine metabolism were 5.0 ± 1.02, 1.35 ± 0.46, 4.81 ± 1.14 µg/mL in human liver microsomes, and 13.69 ± 1.11, 1.19 ± 1.09, 1.69 ± 1.17 µg/mL in rat liver microsomes, respectively. Moreover, imperatorin and isoimperatorin were CYP450s inhibitors with IC_50_ < 10 µM for CYP1A2, 2C9, 2D6, and 3A4. Therefore, this study concluded that Radix *Angelicae dahuricae* could increase ligustrazine plasma concentration and then reinforce its pharmacological effect by inhibiting its metabolism through interference with CYP450s. This could be one mechanism for the synergy between Radix *Angelicae dahuricae* and ligustrazine on migraine treatment.

## 1. Introduction

The liver is the most important site for xenobiotic biotransformation in the body. The cytochrome P450 enzymes (CYP450s) are responsible for more than 90% of marketed therapeutic drug metabolism. Moreover, CYP450 inhibition might lead to an increase in the pharmacokinetic profile in plasma or organs, resulting in the augmentation of therapeutic effect or toxic reactions [1,2]. Thus, CYP450 inhibition might be one mechanism to mediate the synergy of co-administered medicines. 

**Figure 1:**
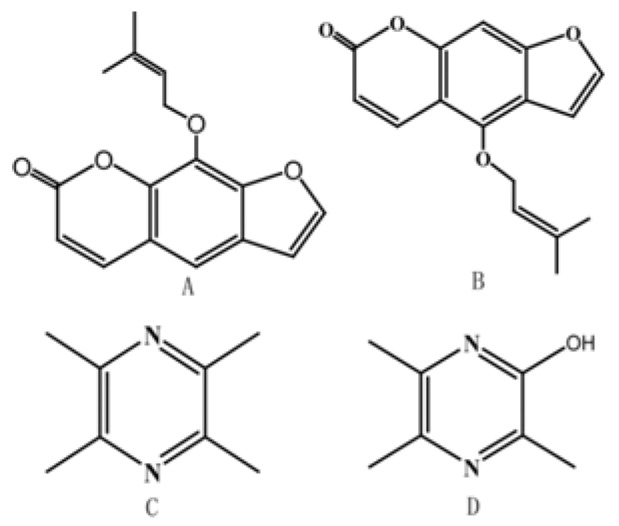
Chemical structures of imperatorin (**A**), isoimperatorin (**B**), ligustrazine (**C**) and hydroxy-ligustrazine (**D**).

Migraine is a kind of paroxysmal neurovascular dysfunction, with the characteristic of repeated attacks of unilateral or bilateral pulsatile severe headaches [3,4]. Combination therapy via medications with similar efficacy have always been applied in migraine treatment. Furthermore, researchers have pointed out that pharmacokinetic synergistic action such as increasing the drug absorption rate or prolonging the drug presence also has benefits for combination therapy in migraine [5]. However, less work has been done in the area of positive pharmacokinetic drug interactions for the treatment of migraine. Radix *Angelicae dahuricae* is the root of *Angelica dahuricae* (Fisch. ex Hoffm.) Benth. et Hook. F. or the root of *Angelica dahuricae* (Fisch. ex Hoffm.) Benth. et Hook. f. var. formosana (Boiss.) Shan et Yuan. It is always co-administered with another medicine [6,7], such as ligustrazine (Figure 1), a synthetic drug with different forms (e.g., tablet and injection) and is used for the clinical treatment of ischemic cerebrovascular disease and migraine in China [8,9,10]. Animal experimental evidence indicates that the main ingredients in Radix *Angelicae dahuricae*, furanocoumarins (such as imperatorin and isoimperatorin, Figure 1), have the potential to increase the pharmacokinetic and therapeutic effects of co-administered drugs [7,11,12,13,14,15]. Moreover, Radix *Angelicae dahuricae* (three times a day for 13 days) was found to significantly decrease the activity of CYP1A2 to 10% of baseline activity in healthy volunteers [16]. Besides, CYP450s, mainly the isoforms of CYP3A, were demonstrated to mediate the metabolism of ligustrazine, while one of the main metabolites was hydroxy-ligustrazine [17,18]. Hence, a metabolism-based synergy between Radix *Angelicae dahuricae* and ligustrazine might exist when Radix *Angelicae dahuricae* was co-administered clinically with ligustrazine or ligustrazine-containing plants.

The present study describes a substantial metabolism-based synergy between total coumarin extract (TCE) of Radix *Angelicae dahuricae* and ligustrazine. TCE could increase the plasma concentration of ligustrazine and then reinforce its pharmacological effect by inhibiting its metabolism through interferences with CYP450 activity. Taken together, these results provide a positive pharmacokinetic drug interaction case in migraine combination therapy study, and also could be a reference for the clinical reasonable combining use of Radix *Angelicae dahuricae* and ligustrazine. The selection of ligustrazine dosage was based on its clinical use [19], while the dosage of TCE was about twice that of Radix *Angelicae dahuricae* in clinical use (2015 Chinese Pharmacopoeia). 

## 2. Results

### 2.1. TCE Enhanced the Anti-Migraine Effects of Ligustrazine 

The rats in the normal group occasionally exhibited head scratching during the observation period. The model group rats displayed frequent head scratching behaviors in the entire observation period (0–180 min after nitroglycerin injection, *p* < 0.05; Table 1). The number of head scratching incidents in the TCE group declined within 60–120 min (*p* < 0.05; Table 1), while it reduced within 60–180 min (*p* < 0.05; Table 1) in the ligustrazine and TCE + ligustrazine groups. Besides, the number of head scratching incidents in the TCE + ligustrazine treatment group was significantly less than that in the ligustrazine treatment group (*p* < 0.05; Table 1).

**Figure 2:**
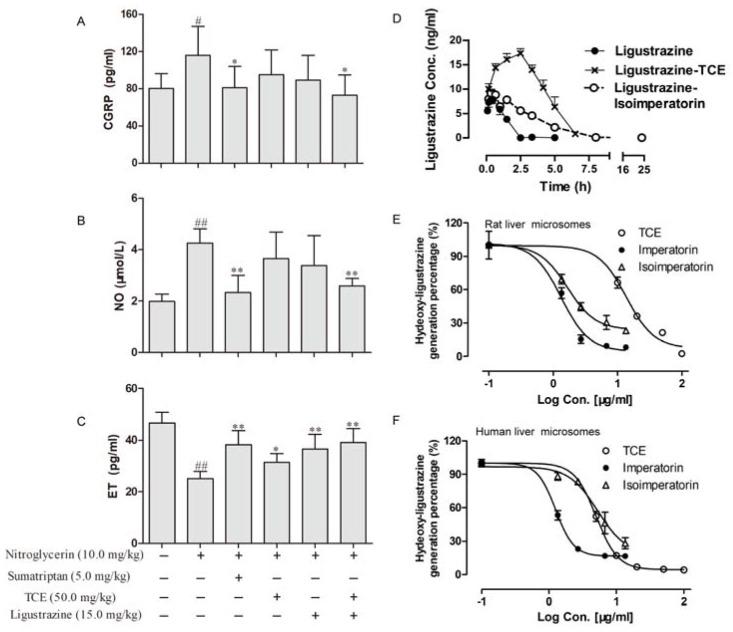
Pharmacodynamic and pharmacokinetic interaction between TCE and ligustrazine (*n* = 6). (**A–C**) The plasma CGRP, serum NO and ET levels in rats with migraine (induced by subcutaneously injected with 10 mg/kg nitroglycerin) after TCE (50.0 mg/kg) alone or treatment with ligustrazine (15.0 mg/kg). (**D**) Pharmacokinetic performance after a single oral dose of 15.0 mg/kg ligustrazine with or without TCE (or isoimperatorin, 50.0 mg/kg) treatment in rats. (**E**, **F**) IC_50_ of TCE, imperatorin, and isoimperatorin for inhibiting ligustrazine metabolism in human (or rat) liver microsomes. TCE: total coumarin extract of *A. dahuricae*, CGRP: calcitonin gene-related peptide, NO: nitric oxide, ET: endothelin. ^#^ *p* < 0.05, ^##^ *p* < 0.01 vs. the control group; * *p* < 0.05, ** *p* < 0.01, vs. the nitroglycerin group; +, presence; −, absence.

When the rats were subcutaneously injected with nitroglycerin (10 mg/kg), the plasma CGRP and serum NO levels were obviously higher than those in the normal group (Figure 2A,B; *p* < 0.05), while the plasma ET level was markedly lower than that in the normal group (Figure 2C; *p* < 0.05). After ligustrazine alone or co-administration treatment with TCE, the plasma CGRP and serum NO significantly decreased only in the TCE + ligustrazine group (Figure 2A,B; *p* < 0.05). However, the ET level significantly increased in TCE, ligustrazine, or their co-administration group (Figure 2C; *p* < 0.05).

### 2.2. TCE (or Isoimperatorin) Increased the Ligustrazine Plasma Concentration in Rats and Inhibited Its Metabolism In Vitro

Pharmacokinetic parameters and performance after a single oral dose of 15 mg/kg ligustrazine with or without TCE (or isoimperatorin) treatment in rats are presented in Table 2 and Figure 2D. Both TCE and isoimperatorin could significantly increase the area under the concentration–time curve (AUC) of ligustrazine (*p* < 0.05) and decrease its clearance (*CL*, *p* < 0.05). Besides, the prolonged mean retention time (MRT) as well as the increased half-life (*t*_1/2_, *p* < 0.05) were also observed in combination therapy groups. However, only TCE could prolong the *T*_max_ time (from 40 to 130 min) and augment the *C*_max_ (from 8.83 to 17.68 µg/mL) of ligustrazine.

When ligustrazine was incubated with TCE, imperatorin, and isoimperatorin, the formation velocity of hydroxy-ligustrazine decreased concentration dependently in human liver microsomes or rat liver microsomes. Besides, IC_50_ were calculated to evaluate their inhibition ability on ligustrazine metabolism. The IC_50_ of TCE, imperatorin and isoimperatorin were 5.0 ± 1.02, 1.35 ± 0.46, 4.81 ± 1.14 µg/mL in human liver microsomes, and 13.69 ± 1.11, 1.19 ± 1.09, 1.69 ± 1.17 µg/mL in rat liver microsomes, respectively (Figure 2E,F). 

### 2.3. Isoimperatorin (or Imperatorin) Inhibited CYP450s Activity and Their Metabolic Information

The IC_50_ of imperatorin and isoimperatorin on recombinant CYP450s is reflected in Table 3. Imperatorin and isoimperatorin displayed medium-to-strong inhibitory effects against the fluorescence substrate for CYP1A2, 2C9, 2D6, and 3A4 (with IC_50_ < 10.0 µM) and weak inhibitory effects on CYP2C19 (with IC_50_ > 15.0 µM). Moreover, the inhibition ability of imperatorin on CYP1A2 and imperatorin (or isoimperatorin) on CYP3A4 was comparable to that of positive control inhibitors (Table 3). 

**Figure 3:**
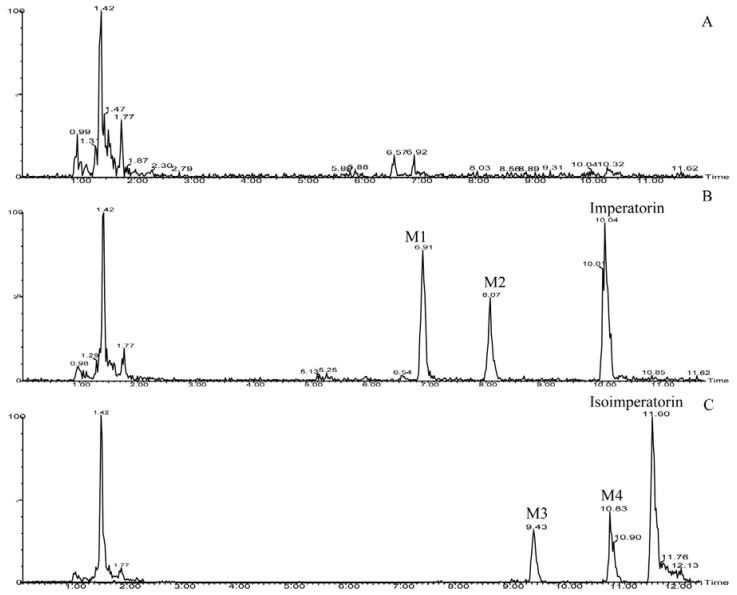
Ultra-performance liquid chromatography–mass spectrometry combined with electrospray ionization (UPLC–ESI–MS/MS) total ion current chromatograms. (**A**) The blank human liver microsomes sample. (**B**) The metabolism sample of imperatorin, with metabolites of M1 and M2. (**C**) The metabolism sample of isoimperatorin, with metabolites of M3 and M4.

**Figure 4:**
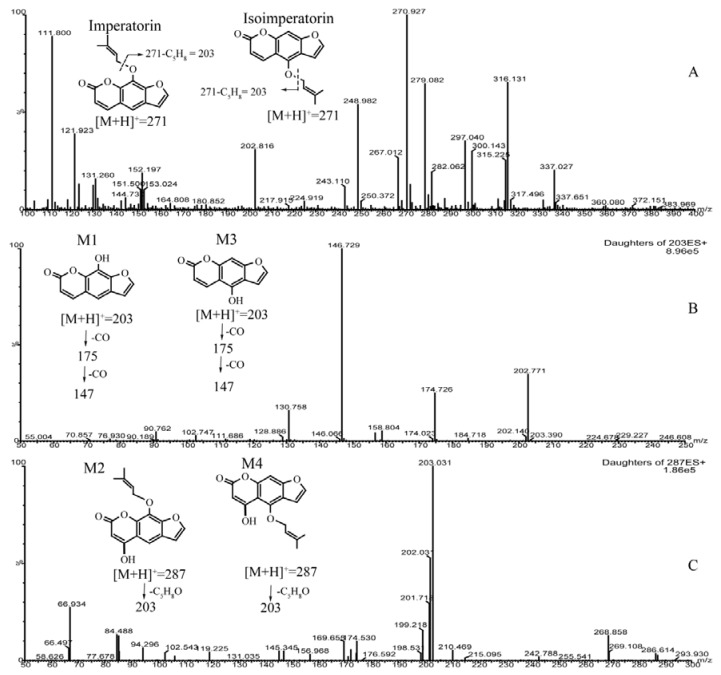
Populated imperatorin and isoimperatorin metabolites in human liver microsomes. (**A**) Imperatorin or isoimperatorin gave the protonated molecule [M + H]^+^ at *m*/*z* 271, and the MS_2_ spectrum showed prominent ions at *m*/*z* 203 ([M + H − C_5_H_8_]^+^). (**B**) M1 and M3 gave [M + H]^+^ at *m*/*z* 203, with MS_2_ spectrum showed prominent ions at *m*/*z* 175 ([M + H − CO]^+^) and *m*/*z* 147([M + H − 2CO]^+^). (**C**) M2 and M4 gave [M + H]^+^ ion at *m*/*z* 287, with MS_2_ spectrum showed prominent ions at *m*/*z* 203([M + H − C_5_H_8_O]^+^).

The total ion current chromatograms of the imperatorin or isoimperatorin metabolism sample and the blank human liver microsome sample are shown in Figure 3. Two new components were observed in the imperatorin and isoimperatorin metabolism samples, respectively, following incubation with human liver microsomes. M1 and M2 were metabolites of imperatorin, while M3 and M4 were metabolites of isoimperatorin. Imperatorin or isoimperatorin gave the protonated molecule [M + H]^+^ at *m*/*z* 271, and the MS_2_ spectrum showed prominent ions at *m*/*z* 203 ([M + H − C_5_H_8_]^+^; as shown in Figure 4). However, for their metabolites, M1 and M3 gave [M + H]^+^ at *m*/*z* 203, and the MS_2_ spectrum showed prominent ions at *m*/*z* 175 ([M + H − CO]^+^) and *m*/*z* 147 ([M + H − 2CO]^+^; as shown in Figure 4). Compared with the fragmentation pathways of imperatorin (or isoimperatorin), M1 and M3 were presumed to be ether linkage hydrolysis metabolites. M2 and M4 gave [M + H]^+^ ions at *m*/*z* 287, which were 16 Da heavier than those of imperatorin (or isoimperatorin). The MS_2_ spectrum showed prominent ions at *m*/*z* 203 ([M + H − C_5_H_8_O]^+^; as shown in Figure 4). Hence, they were presumed to be hydroxylated metabolites of imperatorin and isoimperatorin. 

**Figure 5:**
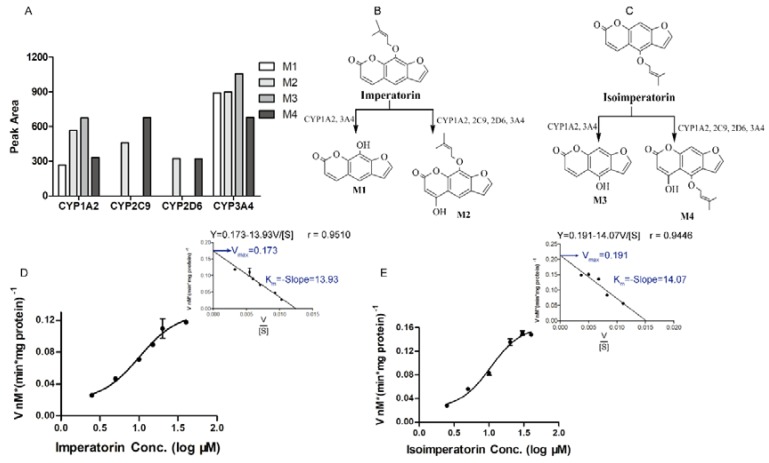
Metabolic pathway and metabolic kinetic constant information of imperatorin and isoimperatorin in human liver microsomes. (**A**) 50 µM Imperatorin (or isoimperatorin) was incubated with recombinant CYP450s, and the relative quantity of metabolite M1, M2, M3 and M4 were determined by the peak area of extract ion chromatograms. (**B**,**C**) Summary of imperatorin and isoimperatorin metabolic pathways. (**D**,**E**) Imperatorin (or isoimperatorin) metabolites formation rate vs. its parent drug Log concentrations (in accordance with the Michaelis–Menten saturation curve) and their *K*_m_ and *V*_max_ in human liver microsomes were calculated from the Eadie–Hofstee plot (inset).

Moreover, when imperatorin (or isoimperatorin) was incubated with recombinant CYP450s, the relative quantity of M1, M2, M3 and M4 were determined by the peak area of extract ion chromatograms (Figure 5A). These results reflected that M1 (or M3) and M2 (or M4) were simultaneously observed in CYP1A2 and CYP3A4 incubation samples. CYP2D6 and CYP2C9 could only metabolize imperatorin (or isoimperatorin) to M2 (or M4). Above all, CYP1A2 and 3A4 might mediate both the ether linkage hydrolysis and hydroxylation reaction of imperatorin/isoimperatorin, while CYP2D6 and 2C9 could only mediate their hydroxylation (Figure 5B,C).

As shown in Figure 5D,E, the formation rate of imperatorin (or isoimperatorin) metabolites increased in a non-linear manner, which in accordance with the Michaelis–Menten saturation curve. The Eadie–Hofstee plot was then applied to calculate their *K*_m_ and *V*_max_. The *K*_m_ and *V*_max_ in human liver microsomes were 13.93 µM and 0.173 nmol ⋅ (min ⋅ mg protein)^−1^ for imperatorin and 14.07 µM and 0.191 nmol ⋅ (min ⋅ mg protein)^−1^ for isoimperatorin, respectively. 

## 3. Materials and Methods

### 3.1. Materials

Imperatorin (purity ≥ 98%), isoimperatorin (purity ≥ 98%), ketoconazole (purity ≥ 99%), atenolol (purity ≥ 98%), and metoprolol (purity ≥ 98%) were obtained from the National Institute for the Control of Pharmaceutical and Biological Products (Beijing, China). Ligustrazine (purity ≥ 98%) was obtained from the Nanjing Zelang Medical Technology Co. Ltd. (Nanjing, China). Hydroxy-ligustrazine (purity ≥ 95%) was a gift from Shandong University, China. Sulfaphenazole, α-naphthoflavone, troglitazone, quinidine (purities ≥ 99%), and β-nicotinamide adenine dinucleotide phosphate (NADPH) were purchased from Sigma Chemical Co. (Saint Louis, MO, USA). The P450-Glo Screening Systems were purchased from Promega Corporation (Madison, WI, USA). Rat liver microsomes, pooled human liver microsomes, and recombinant CYP450 were purchased from the Research Institute for Liver Diseases Co., Ltd. (Shanghai, China). Radix *Angelicae dahuricae* was purchased from Bozhou (Anhui, China). LC-MS grade methanol, ethanol and acetonitrile were provided by Thermo Fisher Scientific, Inc. (MA, USA). H_2_O was of milli-Q grade (Millipore, Billerica, MA, USA). 

### 3.2. Total Coumarin Extract (TCE) of Radix Angelicae Dahuricae Preparation

Radix *Angelicae dahuricae* were purchased from Beijing tongren Hall pharmacy (Beijing, China), and has been identified as the root of *Angelica dahurica* (Fisch. ex Hoffm.) Benth. et Hook. f. by Hua Jin professor in the School of Chinese Materia Medica, Tianjin University of Traditional Chinese Medicine.

The preparation of TCE and its fingerprint have been reported on by our laboratory earlier [15]. In brief, a total of 1 kg Radix *Angelicae dahuricae* was extracted thrice with 8 L of 75% ethanol under reflux for 1.5 h each. The pooled extract was concentrated under reduced pressure at 50 °C. The dry powder was pulverized using macroporous resin as follows: seven times water, eight times 30% ethanol, and eight times 75% ethanol. A solution of 75% ethanol was obtained and evaporated to dryness. This extract was preliminarily analyzed using the Agilent 1200 series high-performance liquid chromatograph (HPLC) instrument (County of Santa Clara, CA, USA). The chromatographic separation was performed using the Agilent Zorbax HC-C18 (4.6 mm × 250 mm × 5 µm) column (Agilent). The mobile phase comprised (A) methanol and (B) pure water with gradient elution: 0–30 min, 40%–90% (A); 30–40 min, 40% (A). The flow rate was 1.0 mL/min, and the ultraviolet wavelength was 310 nm. The major coumarins in Radix *Angelicae dahuricae* extract were quantified, and the percentages were 7.80%, 4.86%, 2.36%, 2.50%, 2.58%, 1.24%, and 8.74% for imperatorin, isoimperatorin, xanthotoxol, 5-hydroxy-8-methoxy psoralen, oxypeucedanin hydrate, bergapten, and cnidilin, respectively. For the detailed HPLC fingerprint chromatogram of TCE of Radix *Angelicae dahuricae*, please refer to a previous study [15]. 

### 3.3. Animals, Treatment, and Sampling

Adult male Sprague–Dawley rats, weighing 200–220 g, were purchased from the Animal Institute of the Tianjin University of Traditional Chinese Medicine (Tianjin, China). The animal approval number of the Experimental Animal Center was SYXK 2013-0003. The test animals were housed in rat cages in a unidirectional airflow room under controlled conditions (20 °C–24 °C; 12-h light/12-h dark cycles, and free access to water). The animals were allowed to acclimatize to the facilities and environment for 7 days before the experiments. The research was conducted in accordance with the internationally accepted principles for laboratory animal use and care of the European Community guidelines (EEC Directive of 1986; 86/609/EEC).

For pharmacokinetic interaction experiments, ligustrazine was administered (15 mg/kg, p.o.) 30 min after treatment with TCE [50 mg/kg, orally (p.o.)] or isoimperatorin (50 mg/kg, p.o.). Serial blood samples were collected in heparinized tubes from the rat orbital sinus under light ether anesthesia at different times after dosing. The blood samples were centrifuged at 2268 g for 10 min, and the plasma fractions were frozen at −20 °C until ultra-performance liquid chromatography–mass spectrometry (UPLC–MS/MS) analysis.

For pharmacodynamic interaction experiments, 36 rats were randomly divided into 6 groups: normal control group, model control group (physiological saline), positive group (Sumatriptan 5.0 mg/kg), TCE group (50.0 mg/kg), ligustrazine group (15.0 mg/kg), and TCE (50.0 mg/kg) + ligustrazine (50.0 mg/kg) group. All groups were orally administered with test compounds containing 2% carboxymethylcellulose sodium (CMC-Na) for 3 days consecutively. On the basis of the previous methods [20], all rats, except those in the normal control group, were subcutaneously injected with nitroglycerin (10 mg/kg) 30 min after the last treatment. The rats in the normal control group were injected with an equivalent volume of vehicle. After model establishment, the numbers of head-scratching incidents were observed continuously using a digital camera (SONY DSC-WX9, Japan) for 3 h. After 4 h of establishing the model, all rats were anesthetized using 1.5% isoflurane. Blood samples were collected from the orbital venous plexus to determine plasma endothelin (ET) and calcitonin gene-related peptide (CGRP) levels using the radioimmunoassay method and serum nitric oxide (NO) level using the colorimetric method.

### 3.4. Ligustrazine Metabolism Interference Study in Liver Microsomes

TCE (5.0, 10.0, 20.0, 50.0, and 100.0 μg/mL), imperatorin (1.35, 2.7, 6.75 and 13.5 μg/mL), or isoimperatorin (1.35, 2.7, 6.75 and 13.5 μg/mL) was incubated with ligustrazine (40.0 μg/mL) in human liver microsomes (2.0 mg/mL) or rat liver microsomes (2.0 mg/mL) for 2 h (37 °C) in the presence of NADPH (1.0 mM) to observe their influence on the ligustrazine metabolism in vitro. The reaction was terminated by adding ice-cold methanol. The contents were vortex-mixed for 30 s and centrifuged at 21,952 g for 15 min. The hydroxy-ligustrazine in the supernatant was analyzed by HPLC.

### 3.5. IC_50_ of Imperatorin or Isoimperatorin on CYP450s Enzymes

The inhibitory effects (in terms of the half-maximal inhibitory concentration, *IC*_50_) of imperatorin or isoimperatorin at concentrations ranging from 0.1 to 200 µM on CYP1A2, 2C9, 2C19, 2D6, and 3A4 were determined. The incubation procedure was based on the product description provided by Promega Corporation. For detailed information, please see Promega Corporation website: https://www.promega.com/resources/protocols/technical-bulletins/101/p450-glo-assays-protocol/. 

### 3.6. Metabolism of Imperatorin or Isoimperatorin in Human Liver Microsomes and Recombinant CYP450s

For *K*_m_ and *V*_max_ determination, imperatorin or isoimperatorin (2.5 μM, 5.0 μM, 10.0 μM, 20.0 μM and 40.0 μM) was incubated with human liver microsomes (1.0 mg/mL) in the presence of NADPH (1.0 mM) for 90 min (37 °C) in a shaking water bath. The reaction was terminated by adding 300 μL of ice-cold methanol, while the residue imperatorin or isoimperatorin in the supernatant was analyzed by HPLC. The calculated parent compound disappearance rates were used to determine the *K*_m_ and *V*_max_. Besides, their metabolites of human liver microsomes or four recombinant CYP450s (CYP1A2, 2C9, 2D6, and 3A4) were identified using UPLC–MS/MS [21,22]. 

### 3.7. Analysis of Samples

#### 3.7.1. Determination of Ligustrazine Concentration

Sample preparation was performed as follows: 0.1 mL of drug-positive plasma was added to bergapten (20 ng, used as an internal standard), and then precipitated using 0.4 mL of organic solvent (methanol:acetonitrile, 1:3). Afterward, each mixture was separated by centrifugation (12,000 *g*, 5 min), and the supernatant fraction was then dried under a stream of nitrogen. The residue was then reconstituted in 50 μL of solvent (methanol:water, 1:1). Finally, 5 μL was injected into the UPLC-MS/MS system. 

The UPLC-MS/MS method was conducted using a Waters Acquity UPLC Sample Manager and a Waters Acquity UPLC Binary Solvent Manager connected to a Waters Quattro Premier XE triple-quadrupole mass spectrometer equipped with a combined electrospray ionization (ESI) probe and Mass Lynx 1.4 software (Waters, MA, USA). An Acquity UPLC BEH C18 column (1.7 µm, 2.1 × 100 mm^2^) was used. The instrument settings were as follows: ESI+; source temperature, 110 °C; desolvation temperature, 400 °C; capillary voltage, 3.5 kV; desolvation N_2_, 650 L/h; cone N_2_, 50 L/h; collision energy, 23 eV for ligustrazine and 20 eV for bergapten; cone voltage, 40 V for ligustrazine and 34 V for bergapten. Multiple reaction monitoring (MRM) mode was chosen for each analyte. The first parent/daughter ion pair of ligustrazine and bergapten was *m*/*z* 137.28→55.3 and *m*/*z* 217.29→202.2, respectively. The solvent system consisted of solvent A (0.1% CH_3_COOH) and solvent B (100% acetonitrile). The gradient was as follows: 0–2.0 min, from 10% to 40% B; 2.0–3.0 min, from 40% to 70% B; 3.0–3.2 min, from 70% to 10% B; and then back to 10% B at 5.0 min for column re-equilibrium prior to the next injection. The flow rate of mobile phase was 0.4 mL/min. The injection volume was 5 µL and the cycle time was 5.5 min/injection. For detailed method validation results of ligustrazine, please refer to Supplement 1 (Figure S1 and Table S1).

#### 3.7.2. Determination of Hydroxy-Ligustrazine Concentration

The HPLC method was conducted using the Waters 600E HPLC system and an Agilent Zorbax HC-C18 column (4.6 mm × 250 mm × 5 µm). The mobile phase consisted of methanol and 0.5% acetic acid (20:80). The wavelength was set at 290 nm. A total of 10 µL of the sample was injected for analysis. The flow rate was set at 0.8 mL/min throughout the analysis, and the column temperature was maintained at 30 °C. For detailed method validation results of hydroxy-ligustrazine, please refer to Supplement 1 (Figure S2 and Table S2).

#### 3.7.3. Determination of Imperatorin or Isoimperatorin Concentration

The HPLC method was conducted using the Waters 600E HPLC system and an ODS-2 Hypersil C_18_ column (5 μm, 250 × 4.6 mm^2^). The mobile phase consisted of 70% acetonitrile and 30% pure water, with absorption wavelength at 285 nm. A total of 10 µL of the sample was injected for analysis. The flow rate was set at 1.0 mL/min throughout the analysis, and the column temperature was maintained at 30 °C. For detailed method validation results of imperatorin or isoimperatorin, please refer to Supplement 1 (Figures S3 and S4 and Tables S3 and S4).

#### 3.7.4. Analysis of Imperatorin and Isoimperatorin Metabolite Using Ultra-Performance Liquid Chromatography–Mass Spectrometry (UPLC–MS/MS)

The UPLC/MS/MS method was conducted using a Waters Acquity UPLC Sample Manager and a Waters Acquity UPLC Binary Solvent Manager connected to a Waters Quattro Premier XE triple-quadrupole mass spectrometer equipped with a combined ESI probe and Mass Lynx 1.4 software (Waters, MA, USA). An Acquity UPLC BEH C18 column (1.7 µm, 2.1 × 100 mm^2^) was used. Instrument settings were as follows: ESI+; source temperature, 110 °C; desolvation temperature, 350 °C; capillary voltage, 3.2 kV; desolvation N_2_, 600 L/h; cone N_2_, 50 L/h; LM resolution 1, 13.5; and HM resolution 1, 13.5. The data acquisition was carried out at *m*/*z* 50–500 Da in the total ion scan mode. The MS/MS spectra were acquired at *m*/*z* 50–300 Da in the daughter ion scan mode. The solvent system consisted of solvent A (0.1% CH_3_COOH) and solvent B (100% acetonitrile). The flow rate was 0.2 mL/min. The use of different HPLC gradients of 10–80% B for 16 min for imperatorin and isoimperatorin after exposure to human liver microsomes was necessary to separate the metabolites from the parent compound.

### 3.8. Data Analysis 

#### 3.8.1. IC_50_ Value Calculation

The IC_50_ value is an indicator of inhibitory effects. According to normal judgment, IC_50_ < 1.0 μM suggests strong inhibitory efficiency, 1.0 μM < IC_50_ < 10.0 μM suggests medium inhibitory efficiency, and IC_50_ > 10.0 μM suggests weak inhibitory efficiency and may have no clinical meaning. The IC_50_ value was calculated using GraphPad Prism (version 4.0) software (GraphPad Software, La Jolla, CA, USA). 

#### 3.8.2. Metabolic Kinetic Constant

GraphPad Prism (version 4.0) software (GraphPad Software, La Jolla, CA, USA) was used in metabolic constant determination. The metabolic rate *V* nmol ⋅ (min ⋅ mg protein)^−1^ was plotted against each Log concentration of the test compound to obtain a Michaelis–Menten saturation curve. Besides, the Eadie–Hofstee plot (*V* plotted against *V*/[S]) was applied to calculate the *K*_m_ and *V*_max_ values. The equation for the Eadie–Hofstee plot was as below. The Y-intercept when *V*/[S] = 0 was *V*_max,_ while the negative slope was *K*_m_.

$$V=V_{\max}-K_{m}\frac{V}{\left[S\right]}$$

*V*: the metabolite formation rate, [*S*]: the parent drug concentration used in the incubation. 

#### 3.8.3. Pharmacokinetic Parameters

The pharmacokinetic parameters of ligustrazine in plasma were calculated by the non-compartmental model using the WinNonlin software (v6.0, Pharsight Corp., Cary, NC, USA).

### 3.9. Statistical Analysis

All data, except IC_50_, were expressed as mean ± standard deviation. IC_50_ was expressed as the mean and 95% confidence interval. SPSS 19.0 statistical software was used for one-way analysis of variance, and a least significance difference post-hoc test was used to compare the mean values. The level of significant difference was set at *p* < 0.05. 

## 4. Discussion

The present study intended to explore a potential drug–drug interaction between TCE and ligustrazine in the treatment of rats with migraine. Results show that the inhibition of ligustrazine metabolism by TCE leads to an increase in ligustrazine concentration in rat plasma. Subsequently, a synergistic action was observed when TCE and ligustrazine were co-administered to treat the migraine in rats. The aforementioned results prove that a pharmacokinetic synergistic action exists in combination therapy with TCE and ligustrazine. 

Migraine is a frequent and challenging condition for practicing neurologists. Therefore, researchers proposed a combination therapy regimen for migraine because its management is generally unsatisfactory [23,24,25]. Moreover, two or more medications known to be efficacious in (episodic) migraine prevention have always been co-administered to provide a synergistic effect. However, in a single small study, the patients were relatively resistant to sumatriptan monotherapy (50 mg oral) but became responsive when 10 mg oral metoclopramide was co-administered, indicating an increase in the absorption of sumatriptan [5,26]. Combination therapies are often known to provide improved effectiveness via pharmacokinetic or pharmacodynamic synergistic action. Moreover, the pharmacokinetic interaction may take place when drugs are absorbed, transported, metabolized or eliminated. The present study provided a paradigm of metabolism-based synergy for TCE and ligustrazine on migraine treatment in rats. Further clinical studies are needed to validate this synergistic effect. Besides, a previous study found that the volatile oil of Radix *Angelicae dahuricae* could ameliorate nitroglycerin-induced migraine in rats [6]. Not surprisingly, the pharmacokinetic and pharmacodynamic synergistic effects might both exist when co-administration of Radix *Angelicae dahuricae* and ligustrazine. Subsequently, the optimal dose regime should be evaluated while combining the use of these two drugs. 

The formulations involving Radix *Angelicae dahuricae* recorded in the 2015 Chinese Pharmacopoeia are up to 10%. Hence, a full understanding of the disposition information of its active ingredients in the liver helps to predict the potential drug–drug interactions between other co-administered drugs. Administration of Radix *Angelicae dahuricae* has the potential to interfere with the pharmacokinetic changes of tolbutamide (substrate of CYP2C9) and diazepam (substrate of CYP2C19) in rats [12]. Luszczki et al. found that imperatorin significantly potentiated the anti-convulsant activity of carbamazepine (substrate of CYP3A4) in mice by a pharmacokinetic increase in total brain carbamazepine [13]. Similarly, the reinforcement of the analgesic effects of corydalis alkaloid in mice by total coumarins of Radix *Angelicae dahuricae* was related to the improvement in the plasma concentration of dl-THP (substrate of CYP2D6) [11]. Moreover, previous studies demonstrated that isoimperatorin and imperatorin might be capable of affecting the metabolic activation of procarcinogens by inhibiting human CYP1A1 and CYP1A2 enzymes [27]. 

Metabolism studies on imperatorin and isoimperatorin have been reported previously. Qiao et al. reported 51 urinary metabolites after an oral administration of imperatorin (80 mg/kg) in rats [28]. Later, Song et al. found 32 metabolites in rat bile after an oral administration of imperatorin (100 mg/kg) [29]. Both the studies have reported the same metabolites of M1 (*m*/*z*, 202) and M2 (*m*/*z*, 286) as found in the present study. Chen et al. have determined 18 metabolites when isoimperatorin incubated with rat liver microsomes [30], and the metabolites of M3 (*m*/*z*, 202) and M4 (*m*/*z*, 286) found in the present study were involved. Even fewer metabolites have been identified; the present study firstly evaluated their metabolic kinetic constant information in human liver microsomes as well as their metabolic pathways in recombinant CYP450s (CYP1A2, 2C9, 2D6, and 3A4). 

Moreover, the inhibition of TCE on hydroxy-ligustrazine formation rates in human liver microsomes (IC_50_ = 5.0 ± 1.02 µg/mL) was markedly stronger than that of rat liver microsomes (IC_50_ = 13.69 ± 1.11 µg/mL). Therefore, it is suggested that Radix *Angelicae dahuricae* might cause greater interference on the plasma level of ligustrazine in humans than in rats. Hence, further studies are needed to investigate the drug interaction between Radix *Angelicae dahuricae* and ligustrazine in healthy volunteers. 

## 5. Conclusions

The present study provides a positive pharmacokinetic drug interaction case in a migraine combination therapy study. The inhibition of ligustrazine metabolism by TCE led to an increase in ligustrazine concentration in rat plasma. Subsequently, a synergistic action was observed when TCE and ligustrazine were co-administered to treat the migraine in rats. Besides, the metabolic kinetic constant information and metabolic pathways of imperatorin and isoimperatorin in human microsomes have also been identified. Taken together, these results could be a reference for the reasonable clinical use of a combination of Radix *Angelicae dahuricae* and ligustrazine. 

## Figures and Tables

**Table 1:** The effects of total coumarin extract (TCE) and ligustrazine on the frequency of head scratching in rats with migrine (*n* = 6).

Group	Dose (mg/kg)	Time after Injection of Nitroglycerin			
		0–30 min	30–60 min	60–120 min	120–180 min
Control		0.80 ± 0.34	1.21 ± 0.67	0.92 ± 0.45	1.01 ± 0.42
Model (nitroglycerin)	10	43.07 ± 5.87 ^##^	47.90 ± 3.87 ^##^	73.96 ± 3.24 ^##^	51.61 ± 2.80 ^##^
Sumatriptan	0.005	39.17 ± 3.92	41.02 ± 5.67	45.30 ± 4.57 **	23.57 ± 4.43 **
TCE	50	46.06 ± 8.23	46.78 ± 4.45	53.24 ± 4.06 **	47.93 ± 4.26
Ligustrazine	15	42.90 ± 8.70	45 36 ± 2.45	50.50 ± 5.47 **	38.40 ± 2.95 **
TCE + ligustrazine	50 + 15	38.20 ± 4.43	44.81 ± 3.72	40.20 ± 4.48 **^&^	28.20 ± 3.05 **^&&^

Mean ± standard deviation (SD) ^##^ *p* < 0.01, compared with control group; ** *p* < 0.01 compared with model group; ^&^ *p* < 0.05, ^&&^ *p* < 0.01 compared with Ligustrazine group. TCE: total coumarin extract of *A. dahuricae*.

**Table 2:** Pharmacokinetic parameters of ligustrazine after solely or co-administration with TCE (or isoimperatorin) in rats (*n* = 10).

	Ligustrazine	Ligustrazine–Isoimperatorin	Ligustrazine–TCE
T_max_ (min)	30	40	130
C_max_ (µg/mL)	8.52 ± 0.87	8.83 ± 0.38	17.68 ± 2.62 **
*t*_1/2_ (min)	35.59 ± 2.54	95.59 ± 5.88 **	90.43 ± 7.96 **
*k_e_* (min^−1^)	0.01947 ± 0.0019	0.00725 ± 0.001 *	0.007 ± 0.0007 **
*CL* (ml/min)	244.19 ± 88.55	15.67 ± 2.33 **	2.38 ± 0.67 **
*MRT* (min)	58.41 ± 0.637	103.6 ± 6.27 *	152.8 ± 16.6 **
AUC _0-__∞_ (µg/mL*min)	43.000 ± 20.000	670.000 ± 180.000 *	4411.2 ± 988.9 **

Each value is the mean ± SD * *p* < 0.05, ** *p* < 0.01 compared with the level of ligustrazine solely. TCE: total coumarin extract of *A. dahuricae*.

**Table 3:** *IC*_50_ of imperatorin and isoimperatorin on CYP450s (*n* = 3).

CYP450 Isoform	*IC*_50_ (µM) (95% Confidence Interval)		
	Imperatorin	Isoimperatorin	Positive Control ^a^
CYP1A2	0.08 (0.052–0.13)	8.26 (5.75–11.88)	0.09 (0.06–0.12)
CYP2C9	4.81 (3.05–7.71)	5.69 (3.21–10.09)	0.14 (0.10–0.18)
CYP2C19	83.34 (0.66–104.85)	19.58 (12.72–30.15)	6.00 (2.76–13.00)
CYP2D6	9.10 (5.328–15.56)	1.56 (0.54–4.5)	0.05 (0.02–0.11)
CYP3A4	1.10 (0.71–1.71)	1.47 (0.99–2.17)	1.38 (0.74–2.56)

^a^ Selective inhibitors for CYP450: α-naphthoflavone for CYP1A2, sulfaphenazole for CYP2C9, quinidine for CYP2D6, troglitazone for CYP2C19, and ketoconazole for CYP3A4.

## Supplementary Materials

The following are available online.

## Abbreviations

CGRP	calcitonin gene–related peptide
ET	endothelin
NO	nitric oxide
NADPH	β-nicotinamide adenine dinucleotide phosphate
TCE	total coumarin extract of Radix *Angelicae dahuricae*
