# Supplementary material for: A Metabolism-Based Synergy for Total Coumarin Extract of Radix Angelicae Dahuricae and Ligustrazine on Migraine Treatment in Rats
Source: Molecules. 2018 Apr 25;23(5):1004. doi: 10.3390/molecules23051004 (PMC6102536; doi:10.3390/molecules23051004)
Supplement: Supplementary file 1 [file molecules-23-01004-s001.pdf]

1. Method validation for ligustrazine

No interference was detected at the retention time for ligustrazine and the internal standard (bergapten) in rat blank plasma samples (Figure S1). The intra- and inter-day precision, accuracy, matrix effect and recovery in plasma samples were satisfactory (Table S1). Inter and intra-day precisions were less than 15%. Recoveries obtained from spiked samples ranged from 50.7% to 62.3%. The matrix effect was between 63.6% and 88.3%. The ligustrazine calibration curve was linear between 20.0 and 1200.0 ng/mL in rat plasma samples. The regression coefficient for the calibration curve was higher than 0.998 (Figure S1D). The lower limit of detection (LOD) and the lower limit of quantitation (LOQ) for ligustrazine were 0.5 ng/mL and 5.0 ng/mL, respectively.

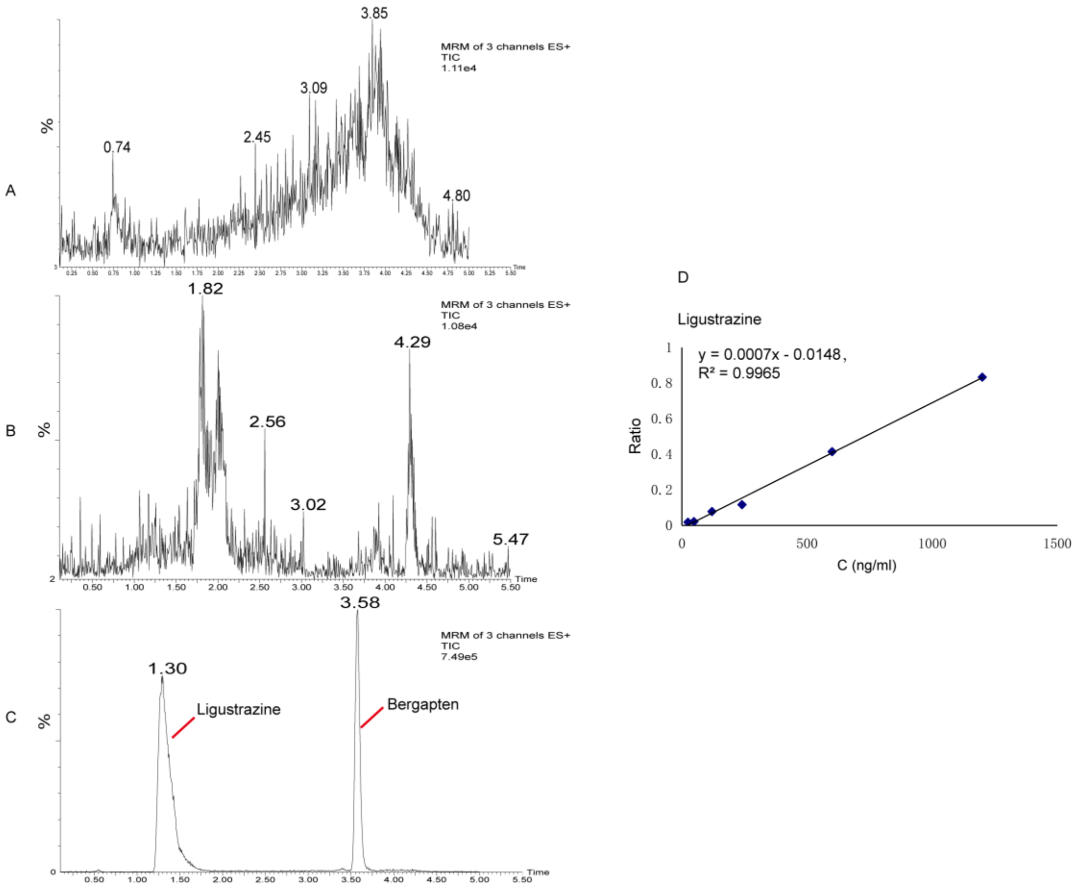

Figure S1 MRM chromatograms and calibration curves of ligustrazine and bergapten. A, blank rat plasma sample; B, blank rat plasma sample spiked with 2% carboxymethylcellulose sodium; C, blank rat plasma sample spike for ligustrazine and bergapten; D, calibration curve of ligustrazine in rat plasma samples.

Table S1Accuracy, intra- and inter-day precision, matrix effect and recovery of the method (n=5）

Compound	Spiked concentration （ng/ml）	Accuracy (%)	Intra-day precision (RSD, %)	Inter-day precision (RSD, %)	Matrix effect (%)	Recovery (%)
ligustrazine	400	102.9±1.2	5.76	4.88	64.9±2.3	55.0±3.1
	1200	94.6±2.6	3.45	4.63	66.5±3.4	50.7±2.8
	6000	107.5±2.4	5.60	5.21	63.6±1.5	57.4±4.6
bergapten	200	98.76±5.3	6.02	4.87	88.3±5.4	62.3±2.3

2. Method validation for hydroxy-ligustrazine

No interference was detected at the retention time of hydroxy-ligustrazine in human liver or rat liver microsome samples (Figure.S2). The intra- and inter-day precision, accuracy and recovery in human (or rats) liver microsome samples were satisfactory (Table. S2). Inter and intra-day precisions were less than 15%. Recoveries obtained from spiked samples ranged from 79.0% to 92.9%. The hydroxy-ligustrazine calibration curves were linear between 20.0 and 1280.0 ng/mL in human liver microsomes, and between 20.0 and 640.0 ng/mL in rats liver microsomes. The regression coefficient for both calibration curves were higher than 0.998 (Figure S2E). The LOD and the LOQ for hydroxy-ligustrazine were 4.0 ng/mL and 10.0 ng/mL, respectively.

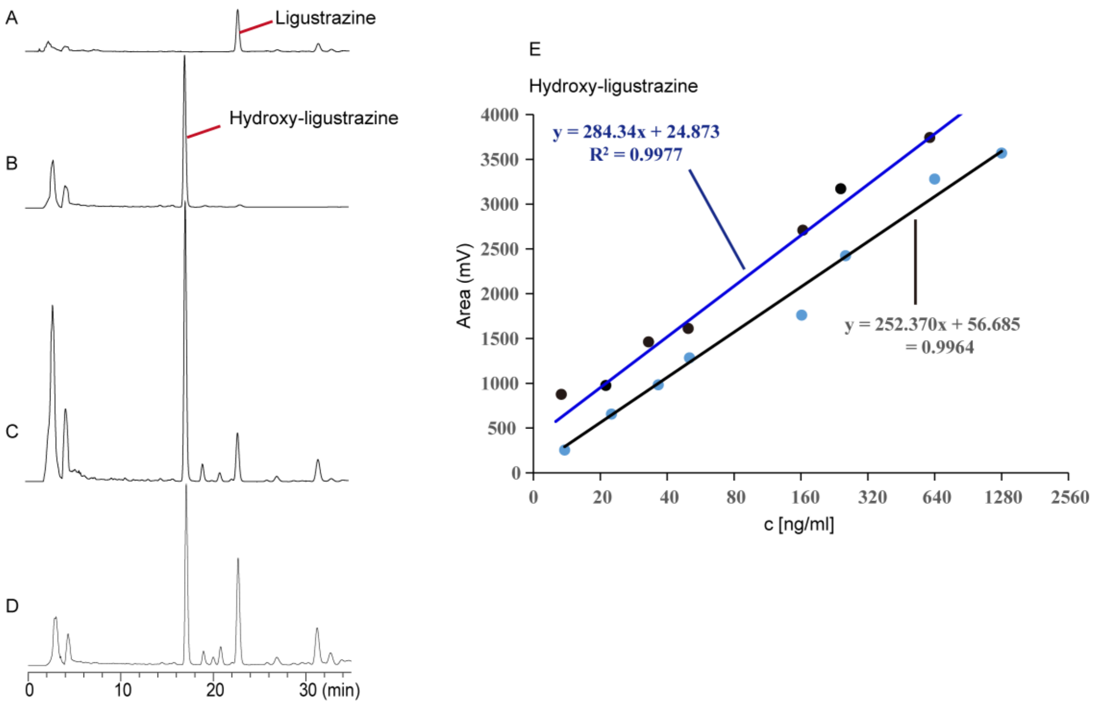

FigureS2. Chromatograms and calibration curves for hydroxy-ligustrazine. A, hydroxy-ligustrazine; B, ligustrazinea; C, blank human liver microsomes sample spike at hydroxy-ligustrazine and ligustrazinea; D, blank rat liver microsomes sample spike at hydroxy-ligustrazine and ligustrazinea. E, calibration curves of hydroxy-ligustrazine in human liver microsomes (black) or in rats liver microsomes (blue).

Table S2 Accuracy, intra- and inter-day precision, recovery of the method (n=5）

Spiked concentration （ng/ml）	Accuracy (%)		Intra-day precision (RSD, %)		Inter-day precision(RSD, %)		Recovery (%)	
	HLM	RLM	HLM	RLM	HLM	RLM	HLM	RLM
640	109.1 ±2.3	89.7 ±3.4	3.8	2.4	5.1	8.4	86.5±8.3	92.2 ±9.0
160	98.6 ±2.5	103.3 ±4.6	0.64	2.5	2.4	2.6	88.7±6.7	87.7 ±4.7
40	90.3± 4.6	88.0 ±6.7	6.9	4.0	9.3	5.4	79.0±6.5	82.3 ±8.6

HLM:human liver microsomes; RLM: rats liver microsomes

3. Method validation for imperatorin

No interference was detected at the retention time of imperatorin in human liver microsomes (Figure S3). The intra- and inter-day precision, accuracy and recovery in

human liver microsome samples were satisfactory (Table S3). Inter and intra-day precisions were less than 15%. Recoveries obtained from spiked samples ranged from 100.62% to 114.51%. The imperatorin calibration curve was linear between 20.0 and 1000.0 ng/mL in human liver microsomes. The regression coefficient for the calibration curve was higher than 0.999 (Figure S3D). The LOD and LOQ for hydroxy-ligustrazine were 2.0 ng/mL and 5.0 ng/mL, respectively.

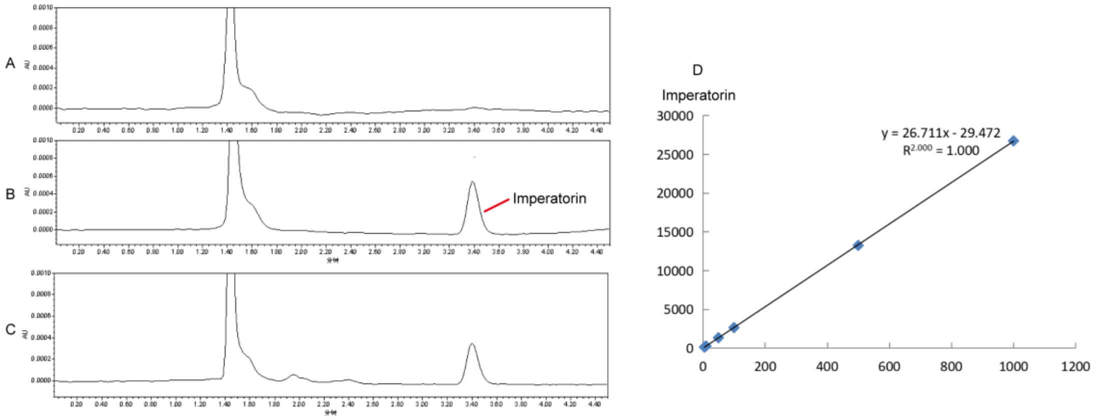

Figure S3. Chromatograms and calibration curve for imperatorin. A, blank solvent (methanol); B, blank solvent spiked with imperatorin; C, blank human liver microsomes sample spiked with imperatorin. D, calibration curve of imperatorin in human liver microsome samples.

Table S3 Accuracy, intra- and inter-day precision and recovery of the method (n=5）

Compound	Spiked concentration （ng/ml）	Accuracy (%)	Intra-day precision (RSD, %)	Inter-day precision (RSD, %)	Recovery (%)
Imperatorin	5.0	100.3±0.93	1.30	0.84	114.51±2.22
	50.0	97.25±4.31	1.43	0.83	100.62±3.22
	500.0	102.4±2.02	3.70	0.45	101.34±0.49

4. Method validation for isoimperatorin

No interference was detected at the retention time of isoimperatorin in human liver microsomes (Figure S4). The intra- and inter-day precision, accuracy and recovery in human liver microsomes samples were satisfactory (Table S4). Inter and intra-day precisions were less than 15%. Recoveries obtained from spiked samples ranged from 98.54% to 110.63%. The isoimperatorin calibration curve was linear between 20.0 and 500.0 ng/mL in human liver microsomes. The regression coefficient for the calibration curve was higher than 0.998 (Figure S4D). The LOD and the LOQ for

isoimperatorin were 2.0 ng/mL and 5.0 ng/mL, respectively.

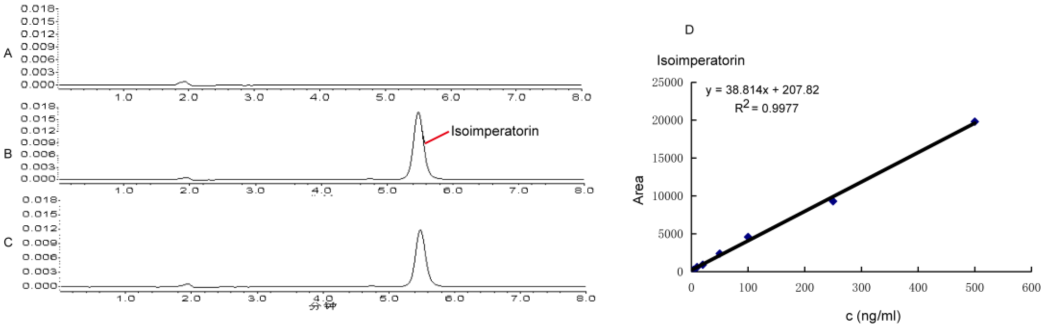

Figure S4. Chromatograms and calibration curve for isoimperatorin. A, blank solvent (methanol); B, blank solvent spiked with isoimperatorin; C, blank human liver microsomes sample spiked with isoimperatorin. D, calibration curve of isoimperatorin in human liver microsome samples.

Table S4 Accuracy, intra- and inter-day precision and recovery of the method (n=5）

Compound	Spiked concentration （ng/ml）	Accuracy (%)	Intra-day precision (RSD, %)	Inter-day precision (RSD, %)	Recovery (%)
Isoimperatorin	20.0	95.25 ± 2.03	2.43	1.71	110.63±5.03
	100.0	96.0±1.10	1.09	0.83	97.24±3.93
	400.0	100.3±4.31	2.38	2.04	98.54±6.94
